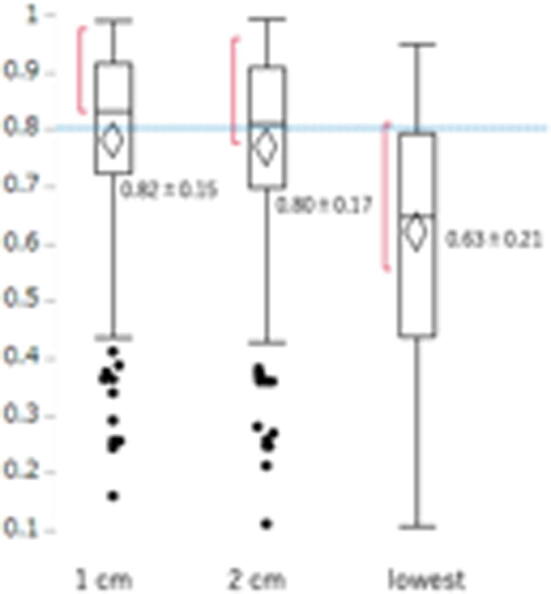# Comparison of diagnostic performance in on-site based CT-derived fractional flow reserve measurements

**DOI:** 10.1016/j.ijcha.2021.100815

**Published:** 2021-06-11

**Authors:** Yui O. Nozaki, Shinichiro Fujimoto, Chihiro Aoshima, Yuki Kamo, Yuko O. Kawaguchi, Kazuhisa Takamura, Ayako Kudo, Daigo Takahashi, Makoto Hiki, Yoshiteru Kato, Iwao Okai, Tomotaka Dohi, Shinya Okazaki, Nobuo Tomizawa, Kanako K. Kumamaru, Shigeki Aoki, Tohru Minamino

**Affiliations:** a Department of Cardiovascular Biology and Medicine, Juntendo University Graduate School of Medicine, Tokyo, Japan; b Department of Radiology, Juntendo University Graduate School of Medicine, Tokyo, Japan; c Japan Agency for Medical Research and Development-Core Research for Evolutionary Medical Science and Technology (AMED-CREST), Japan Agency for Medical Research and Development, Tokyo, Japan

**Keywords:** Coronary CT angiography, Fractional flow reserve, Fluid structure interaction, Lesion-specific ischemia

## Abstract

**Background:**

Computed tomography fractional flow reserve (CT-FFR), which can be acquired on-site workstation using fluid structure interaction during the multiple optimal diastolic phase, has an incremental diagnostic value over conventional coronary computed tomography angiography (CCTA). However, the appropriate location for CT-FFR measurement remains to be clarified.

**Method:**

A total of 115 consecutive patients with 149 vessels who underwent CCTA showing 30–90% stenosis with invasive FFR within 90 days were retrospectively analyzed. CT-FFR values were measured at three points: 1 and 2 cm distal to the target lesion (CT-FFR_1cm, 2cm_) and the vessel terminus (CT-FFR_lowest_). The diagnostic accuracies of CT-FFR ≤ 0.80 for detecting hemodynamically significant stenosis, defined as invasive FFR ≤ 0.80, were compered.

**Result:**

Fifty-five vessels (36.9%) had invasive FFR ≤ 0.80. The accuracy and AUC for CT-FFR_1cm_ and _2cm_ were comparable, while the AUC for CT-FFR_lowest_ was significantly lower than CT-FFR_1cm_ and _2cm_. (lowest/1cm, 2 cm = 0.68 (95 %CI 0.63–0.73) vs 0.79 (0.72–0.86, p = 0.006), 0.80 (0.73–0.87, p = 0.002)) The sensitivity and negative predictive value of CT-FFR_lowest_ were 100%. The reclassification rates from positive CT-FFR_lowest_ to negative CT-FFR_1cm_ and _2cm_ were 55.7% and 54.2%, respectively.

**Conclusion:**

The diagnostic performance of CT-FFR was comparable when measured at 1-to-2 cm distal to the target lesion, but significantly higher than CT-FFR_lowest_. The lesion-specific CT-FFR could reclassify false positive cases in patients with positive CT-FFR_lowest_, while all patients with negative CT-FFR_lowest_ were diagnosed as negative by invasive FFR.

## 1 Introduction

Several techniques for computing fractional flow reserve (FFR) based on images acquired from coronary computed tomography angiography (CCTA) have been developed [1], [2], [3], [4], [5], [6], demonstrating incremental diagnostic value in large-scale multicenter studies. However, the specificity and positive predictive value (PPV) tended to be low compared to the sensitivity and negative predictive value (NPV) [4], [5], [6]. This may lead to overdiagnosis and overtreatment for revascularization based on CT-derived FFR guided management. In previous validation studies [4], [5], CT-derived FFR was measured at the same location as the invasive FFR pressure wire. One of the methods for CT-derived FFR (CT-FFR) calculates coronary flow and pressures by accounting for the shape, movement, cross-sectional area, and changes in volume of the coronary artery using fluid structure interaction during the multiple optimal diastolic phases of the cardiac cycle on 320-row area detector CT [7], [8]. The clinical validation of CT-FFR has been demonstrated by comparison with invasive FFR values [9], [10], however, considering putting CT-FFR to practice use as a standalone diagnostic modality in the future, no unified view has been obtained so far about the appropriate location for measuring CT-FFR in a target vessel. The aim of this study was to investigate the diagnostic performance and characteristics of CT-FFR using this algorithm with 30–90% diameter clinical stenosis in various measurement positions, for comparison with invasive FFR as the reference standard.

## 2 Methods

### 2.1 Study design and population

This was a single-center retrospective study. The subjects were 115 consecutive patients (149 vessels) who underwent CCTA showing intermediate stenosis (range 30–90%) of at least one major epicardial vessel measuring ≥1.8 mm in diameter on CCTA, and subsequent invasive FFR within 90 days from CCTA, between December 2015 to March 2020. Patients who had previously undergone revascularization (PCI and/or CABG) were excluded. There was no one who was excluded with poor image quality. The study protocol conformed to the Declaration of Helsinki and was conducted in compliance with the institutional ethics committee guidelines. Patients informed consent was waived due to the retrospective study design.

### 2.2 CCTA acquisition and interpretation

Patients with a pre-scan heart rate ≥60 beats per minute (bpm) were given 20–40 mg of metoprolol orally. If the heart rate remained ≥61 bpm after 1 h, they were given intravenous landiolol (0.125 mg/kg) (Corebeta; Ono Pharmaceutical, Tokyo, Japan). Patients in whom beta-blockers were contraindicated due to severe aortic stenosis, symptomatic heart failure, bronchial asthma, or advanced atrioventricular block, did not receive these treatments. All patients received 0.6 mg nitroglycerin sublingually (Myocor spray; Toa Eiyo, Tokyo, Japan). Patient preparation and CT scanning were performed based on the Society of Cardiovascular Computed Tomography gulidline [11].

All patients were scanned using a 320-row CT (Aquilion ONE ViSION Edition^TM^ or Genesis Edition^TM^; Canon Medical Systems Corporation, Otawara, Japan). Scanning was performed at a tube voltage of 100 kVp except for patients whose body mass index exceeded 30 kg/m^2^, who were scanned at 120 kVp. The gantry rotation time was 275 ms, and the tube current was 430 to 900 mA. The mean tube current was determined with automatic exposure control target at a standard deviation (SD) of 22. The craniocaudal range was selected from 200 rows (100 mm) to 320 rows (160 mm), to include the entire coronary tree. The contrast agent iopamidol (Iopamiron 370 mg Bayer AG, Leverkusen, Germany) was injected for 12 s at 18 mg I/Kg/s (Dual Shot GX7; Nemoto Kyorindo Co, Ltd, Tokyo, Japan) followed by 30 ml of saline at the same injection rate. A single-heartbeat scan with prospective electrocardiogram gating was performed with a phase window of 70–99% of the R-R interval to cover the entire diastolic phase. For each patient, the phase with minimum artifacts was determined on CT console. Additionally, images of 70%, 80%, 90%, and 99% of the R-R interval were reconstructed to calculate CT-FFR. The slice thickness was 0.5 mm, and the increment was 0.25 mm. Images were reconstructed by using a ‘‘medium soft tissue’’ kernel (FC04) with an iterative reconstruction algorithm (Adaptive Iterative Dose Reduction 3-Dimentional processing, AIDR3D; Canon Medical Systems Corporation).

Images were transferred to a workstation (Ziostation; Ziosoft Inc., Tokyo, Japan) for visual stenosis analysis. Coronary artery segments with a minimum diameter of 1.8 mm were evaluated. The ratio of the stenotic lumen to the normal vessel diameter proximal or distal to the stenosis was obtained, and the degree of stenosis was determined. Measurements were made in the angle showing the narrowest degree of stenosis using a curved planar reconstruction image.

### 2.3 CT-FFR analysis and measurement

**Fig. 1:**
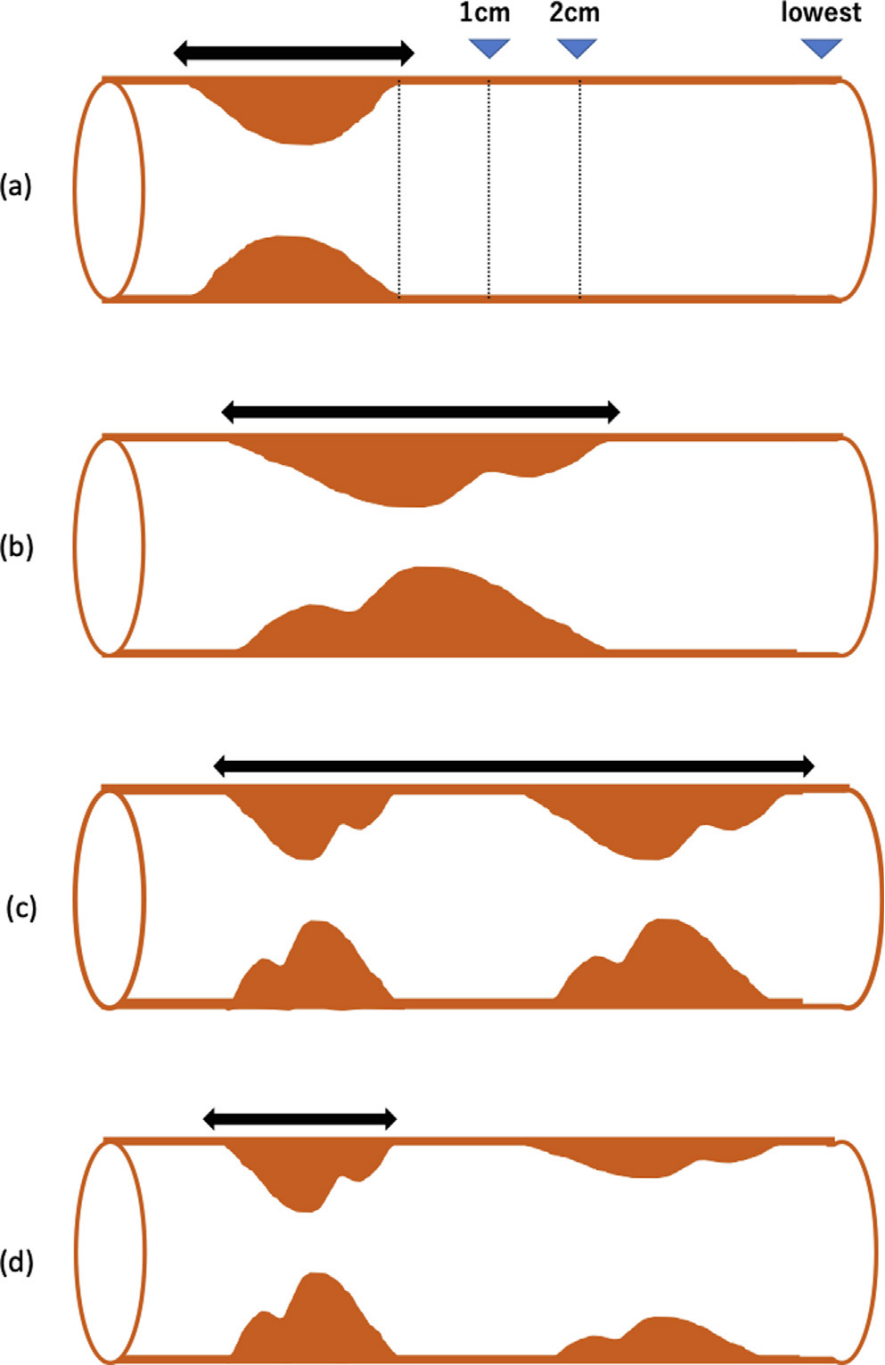
Examples of a target lesion and each measurement: (a) the CT-FFR values at 1 and 2 cm distal to the end of the target lesion, and at the target vessel terminus were evaluated. (b) When the target lesion was sequential, we defined the target as shown by the black line arrow. (c) When the target lesion with ≥30% stenosis was divided into parts, we defined the target as shown the black line arrow. (d) When there was a stenosis <30% distal to a target lesion with ≥30% stenosis, we did not consider the small stenosis as a target.

CT-FFR analysis was performed on a workstation (Vitrea version V7.2; Vital Images In, Minnetonka, Minn). The centerline and the luminal contour of the three major coronary arteries were automatically selected, and manual adjustment was performed when necessary. After the procedure, CT-FFR was calculated using the structural and fluid analysis software (Canon Medial Systems Corporation), which allows the computation of CT-FFR values at any selected points of the coronary tree. CT-FFR was calculated according to the Hierarchical Bayes & Markov-Chain Monte Carlo Method, in consideration of changes in the shape, movement, cross-sectional area and volume of the coronary artery determined using several optimal cardiac phases to acquire 70–99% of the cardiac phase [7], [8]. Hierarchical Bayes & Markov-Chain Monte Carlo Method are applied to determine the analysis conditions. Further, on-site analysis was performed by calculating the 1D computational fluid dynamics. All procedures were performed by a skilled analysist who had >50 h of experience in training with the software and who was blinded to the invasive angiography and invasive FFR results. The coronary severity and three parameters were calculated by consensus of three experienced cardiovascular imagers who were blinded to clinical data as follows: the lowest CT-FFR value at the distal end of the target coronary vessel (CT-FFR_lowest_), and the CT-FFR values at 1 and 2 cm distal to the end of the stenotic lesion in the 30–90% range (CT-FFR_1cm, 2cm_). These parameters were all computed locally on a regular workstation. Fig. 1 shows the details of how each parameter was defined. If a CT-FFR value could not be calculated due to the location being distal to the distal vessel tip, the value of CT-FFR_lowest_ was substituted. (i.e., CT-FFR_2cm_ = CT-FFR_lowest_) (1 cm; 4 cases, 2 cm; 9 cases)

### 2.4 ICA and FFR techniques

Invasive coronary angiography (ICA) and FFR were performed according to standard clinical practice. Percent stenosis was calculated for the narrowest degree of stenosis. FFR was performed at the judgement of the operator with a 0.014-inch pressure guide wire (Verrata Pressure Guide Wire, Volcano Corp., San Diego, CA or Pressure Wire Certus, St Jude Medical System, Uppasala, Sweden). Hyperemia was obtained after administration of intracoronary isosorbide dinitrate (0.5–1.0 mg) and intracoronary papaverine hydrochloride (12 mg for left coronary, 8 mg for right coronary) or intravenous adenosine triphosphate (140 μg/kg/min). FFR was calculated automatically by diving the mean diastole coronary pressure and the mean aortic pressure during hyperemia. The pressure wire was inserted as far as possible, although a target lesion was proximal to a vessel. FFR was considered diagnostic of ischemia at a threshold of ≤0.80.

### 2.5 Statistical analysis

Numerical data was expressed as the mean ± standard deviation for normally distributed variables, as medians with interquartile ranges for non-normally distributed variables, and as numbers of cases (and percentages) per group for categorical variables. The incremental diagnostic prediction ability was calculated using receiver operating characteristic (ROC) analysis with area under curve (AUC), and accuracy, sensitivity, specificity, positive predictive value (PPV) and negative predictive value (NPV) with their corresponding 95% confidence intervals (95% CIs). The AUCs were compared using the method of DeLong et al. [12] values at p < 0.05 were considered as significant. Computations were performed using JMP pro 14.2 (SAS institute INC., Cary, NC, USA), and R 4.0.2 (R Foundation for statistical Computing, Vienna, Austria) software.

## 3 Results

### 3.1 Baseline characteristics

A total of 115 patients were enrolled. Their mean age was 67.8 ± 9.4 years old, and 66% (74/115) of patients were male. The mean heart rate on imaging was 59.1 ± 0.7 bpm. Nitrates were used in all patients. The mean effective CCTA dose was 2.5 ± 1.2 mSv, and the tube voltage was set at 120kVp for 12 patients. The characteristics of patients and CT scans are summarized in Supplemental Tables 1 and 2, respectively. Invasive FFR and CT-FFR were calculated in 149 vessels: RCA, 33; LAD, 85; LCX, 31. Fifty-five (36.9%) had invasive FFR ≤ 0.8; the mean value of invasive FFR was 0.83 ± 0.10. The mean values of CT-FFR_1cm_, _2cm_ and _lowest_ were 0.82 ± 0.15, 0.80 ± 0.17 and 0.63 ± 0.21 respectively. (Supplemental Fig. 1) Vessel characteristics are shown in Supplemental Table 3.

### 3.2 Diagnostic accuracy of CT-FFR and CCTA

**Fig. 2:**
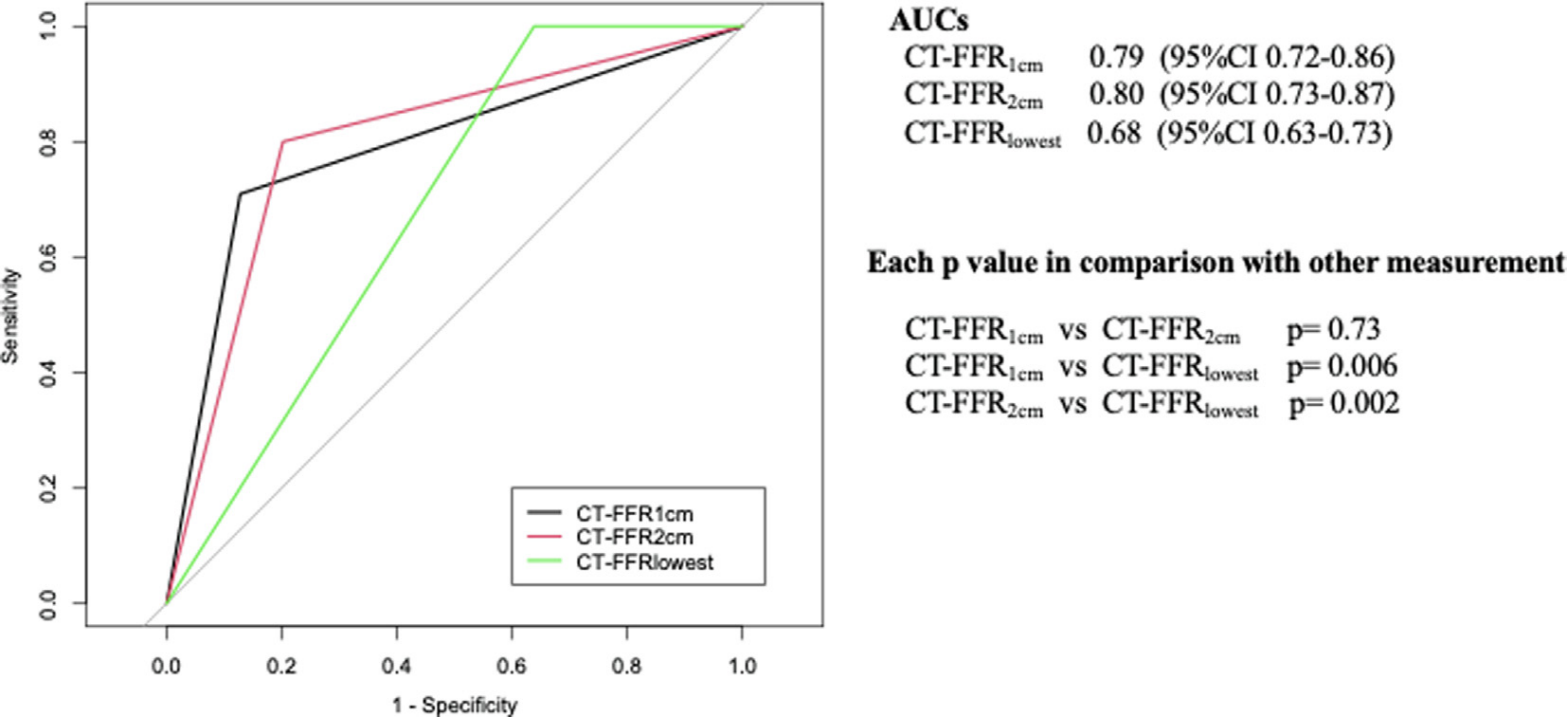
AUC for the characteristics in CT-FFR measured at each point (CT-FFR_1cm, 2cm_ and _lowest_) in predicting invasive FFR ≤ 0.80 per vessel. The AUCs to predict invasive FFR ≤ 0.80 on a per-vessel basis were significantly higher at CT-FFR_1cm_ and _2cm_ than CT-FFR_lowest_ (p < 0.05 in each case.). AUC; area under the curve. Values are mean with 95% confidence intervals.

Table 1 shows the measurements of diagnostic performance (sensitivity, specificity, PPV, NPV and accuracy) used to detect hemodynamically-significant stenosis defined as invasive FFR ≤ 0.80. The values of sensitivity and NPV increased gradually with distance, and finally reached 100% in CT-FFR_lowest_. The reclassification rates from positive CT-FFR_lowest_ to negative CT-FFR_1cm_ or _2cm_ were 55.7% (64/115) and 54.2% (52/115) respectively, while 75.0% (48/64) and 78.8% (41/52) of these vessels were diagnosed as negative by invasive FFR. Fig. 2 shows the ROC curves of CT-FFR_1cm, 2cm_ and _lowest_ in predicting ischemia during invasive FFR. The values of AUCs were 0.79 (95 %CI 0.72–0.86), 0.80 (0.73–0.87), and 0.68 (0.63–0.73) respectively. The diagnostic performance was comparable between CT-FFR_1cm_ and _2cm_, however, the AUC for CT-FFR_lowest_ was significantly lower than CT-FFR_1cm_ and _2cm_. (lowest/1cm, 2 cm = 0.68 vs 0.79 (p = 0.006), and vs 0.80 (p = 0.002)).

**Table 1:** Comparison between various locations of measurements for CT-FFR to identify invasive FFR ≤ 0.80 on per vessel.

CT-FFR	Sensitivity (%) (95% CI)	Specificity (%) (95% CI)	PPV (%) (95% CI)	NPV (%) (95% CI)	Accuracy (%) (95% CI)
1 cm	70.9 (61.7–78.1)	87.2 (81.8–91.5)	76.5 (66.5–84.3)	83.7 (78.5–87.7)	81.2 (74.4–86.5)
2 cm	80.0 (70.6–87.2)	79.8 (74.3–84.0)	69.8 (61.6–76.2)	87.2 (81.2–91.8)	79.9 (72.9–85.2)
lowest	100 (94.2–100)	36.2 (32.8–26.8)	47.8 (45.1–47.8)	100 (90.7–100)	59.7 (55.5–59.7)

PPV; positive predictive value NPV; negative predictive value CI; confidence intervals.

## 4 Discussion

For patients with 30–90% diameter stenotic disease in at least one major vessel, the main findings of our study were that: (1) CT-FFR measured at 1-to-2 cm distal to the target lesion had higher discrimination of ischemia than CT-FFR_lowest_, the latter of which may have limited value for clinical decision-making. The best discrimination of ischemia was not observed between the lesion-specific CT-FFR (CT-FFR_1cm_ and _2cm_); (2) for CT-FFR_lowest_, the sensitivity and NPV were significantly higher but the specificity and PPV were lower; and (3) the lesion-specific CT-FFR could reclassify false positive cases in patients with positive CT-FFR_lowest_, while increasing false negative.

We evaluated CT-FFR at three locations of CT-FFR: 1 and 2 cm distal to the farthest target lesion with diameter ≥1.8 mm, and the target vessel distal terminus. CCTA generally overestimates the degree of coronary stenosis and underestimates vascular diameter, due to imaging artifact by calcification and the spatial resolution. This may cause the specificity and PPV to have lower diagnostic performance than sensitivity or NPV [13]. In our study, the sensitivity and NPV for CT-FFR_lowest_ were 100%, suggesting that we can defer revascularization when the CT-FFR_lowest_ value is classified as negative. This result may also be related to the lower prognostic ability of specificity and PPV. One of the reasons is that there is a gradual decrease in both CT-derived FFR and invasive FFR value with distance, even without stenosis, due to pressure loss by frictional losses according to Poiseulle’s equation [14]. The influence is reportedly more significant in CT-derived FFR than invasive FFR [15].

Although conventional invasive FFR assessment recommends measuring pressure 2-to-3 cm (or 5–10 times the proximal vessel reference diameter) distal to a target lesion [16], there is no consensus about the best location to measure on-site CT-FFR for clinical application. Using the FFR_CT_ algorithm, the most widespread standard developed by HeartFlow Inc. (Redwood City, Calif), recent studies have shown that 35–44% of patients with stable coronary artery disease and lowest FFR_CT_ as positive were reclassified as negative when the FFR_CT_ measurement point was 1-to-2 cm distal to a stenosis [17], [18]. As a recent study also highlighted that the diagnostic performance of FFR_ct_ measured at 1-to-2 cm distal to the stenosis was significantly higher than that of far-distal segments [19], it is now recommended to measure FFR_CT_ 1-to-2 cm distal to the end of a focal stenosis [20]. This is the first study, using the CT-FFR algorithm, which demonstrated the diagnostic performance of CT-FFR was significantly better when measured at 1-to-2 cm distal to a target stenosis than measured at the vessel terminus. In our study, 55.7% (64/115) and 54.2% (52/115) of patients with positive CT-FFR_lowest_ were reclassified as negative for CT-FFR_1cm_ and _2cm_, respectively. However, among these reclassified groups, 25.0% (16/64) and 21.2% (11/52) were positive by invasive FFR. These false positive cases shown in the negative lesion-specific CT-FFR could help patients with intermediate stenosis avoid further investigation, while a certain number of patients would be classified to false negative cases, using the lesion-specific CT-FFR.

Invasive FFR is generally evaluated using the value measured at distal to a target vessel from the viewpoint of evaluating ischemia in the myocardial region where the target vessel is perfused. However, false positives and unnecessary revascularization may increase, if CT-FFR is evaluated at the farthest target vessel. In contrast, if CT-FFR is evaluated distal to a target lesion, patients requiring revascularization might be missed when employing a strategy that takes invasive FFR as the reference.

One observational single-center study reported that the intermediate follow-up clinical outcomes were favorable in patients with terminal FFR_CT_ ≤ 0.80 treated with optimal medical therapy (OMT) [18]. In this study, FFR_CT_ was defined as positive for values ≤0.80 at 2-to-3 cm distal to a focal stenosis (lesion-specific ischemia) or at the vessel terminus with a gradual decline (distal vessel positivity). Although the risk of nonfatal MI in the FFR_CT_ ≤ 0.80 + OMT group was significantly higher than that in the FFR_CT_ ≤ 0.80 + ICA group, all patients who were treated medically with FFR_CT_ ≤ 0.80 and eventually developed nonfatal MI had lesion-specific ischemia [18]. We hypothesize that patients with negative CT-FFR_1-2cm_ might have avoided ICA or revascularization when treated with OMT. When CT-FFR_1-2cm_ is positive, ICA is basically recommended. In the case of negative CT-FFR_1-2cm_, CT-FFR_lowest_ should be also evaluated. OMT is recommended with negative CT-FFR_lowest_, while, if CT-FFR_lowest_ is positive, the pre-test probability and/or clinical background (i.e.; proximal in a target vessel, complicated or bifurcation lesion, multivessel disease, typical chest pain) are taken into consideration before determining the strategy of OMT or ICA. Further studies are needed to access the optimal measurement location in terms of CT-FFR as a standalone diagnostic modality.

## 5 Limitations

Our study has several limitations. First, this was a single-center retrospective study with a small number of subjects and vessels, and therefore careful interpretation is needed. Second, there were some duplicated measurements in case where the farthest stenotic lesion was distal to the intended measurement location (i.e., CT-FFR_1cm_.). We substituted the CT-FFR_lowest_ value in these cases. However, a CT-FFR value measured at terminus vessel sometimes reaches 1–2 cm proximal to a target lesion in real-world clinical practice. Third, judgement on whether to perform invasive FFR was made by the operator during ICA. Moreover, the location for the invasive FFR pressure wire also depended on the operator; the distance from the farthest stenotic lesion to FFR pressure wire was 3.7 cm (95 %CI 3.3–4.2). Forth, as our previous study reported [9], CACS is one of the factors which weakens the diagnostic accuracy in CT-FFR. In this study, we did not analyze the comparison between high and low CACS. Fifth, this study did not consider that a patient background, such as typical angina, echocardiogram data, and the presence of microvascular involvement, might reflect the diagnostic accuracy. In this study, although per-vessel ischemia was simply analyzed and compared, using CT-FFR algorithm, physicians usually decide the clinical strategy with consideration of clinical background.

## 6 Conclusion

The diagnostic performance of on-site workstation-based CT-FFR, which is one of the techniques for computing FFR based on images acquired from CCTA, against invasive FFR as the reference standard, was significantly higher when measured at 1-to-2 cm distal to the end of the farthest stenosis than measured at the vessel terminus. Evaluations made distal to target lesions (1-to-2 cm) may reclassify false positive cases, while increasing false negatives. All patients in whom the lowest CT-FFR was classified as negative were also diagnosed as negative by invasive FFR.

## CRediT authorship contribution statement

**Yui O. Nozaki:** Conceptualization, Methodology, Formal analysis, Data curation, Writing - original draft, Visualization. **Shinichiro Fujimoto:** Conceptualization, Methodology, Formal analysis, Data curation, Writing - original draft, Visualization. **Chihiro Aoshima:** Investigation, Resources. **Yuki Kamo:** Data curation. **Yuko O. Kawaguchi:** Data curation. **Kazuhisa Takamura:** Data curation. **Ayako Kudo:** Data curation. **Daigo Takahashi:** Data curation. **Makoto Hiki:** Data curation. **Yoshiteru Kato:** Data curation. **Iwao Okai:** Data curation. **Tomotaka Dohi:** Data curation. **Shinya Okazaki:** Data curation. **Nobuo Tomizawa:** Data curation. **Kanako K. Kumamaru:** Project administration. **Shigeki Aoki:** Writing - review & editing. **Tohru Minamino:** Writing - review & editing.

## Declaration of Competing Interest

The authors declare the following financial interests/personal relationships which may be considered as potential competing interests: Dr. Fujimoto has a research agreement with Canon Medical Systems Corporation that is related to this study. This research was supported by Canon Medical Systems Corporation. All other authors report no conflicts to the work.

## Figures and Tables

**Supplementary figure 1:**