# Supplementary material for: Comparison of diagnostic performance in on-site based CT-derived fractional flow reserve measurements
Source: Int J Cardiol Heart Vasc. 2021 Jun 11;35:100815. doi: 10.1016/j.ijcha.2021.100815 (PMC8215214; doi:10.1016/j.ijcha.2021.100815)
Supplement: Supplementary data 1 [file mmc1.docx]

**Supplemental Table 1.** Patients Characteristics

|  | **Overall**  **n = 115** |
| --- | --- |
| Gender (M/F) | 76/39 |
| Age (years) | 67.8±9.4 |
| Body mass index (Kg/m^2^) | 24.1±3.2 |
| Hypertension (%) | 74 (64.3) |
| Hyperlipidemia (%) | 87 (75.7) |
| Diabetes mellitus (%) | 57 (49.6) |
| Smoking (none/former/current) | 44/53/18 |
| CACS [25th, 75th orecentiles] | 310.6 [81.1, 786.5] |

CACS; coronary calcium score

**Supplemental Table 2.** Scan Characteristics

|  | **Overall**  **n = 115** |
| --- | --- |
| Heart rate (bpm) | 59.1±0.7 |
| Nitrates administered (%) | 100 |
| β blocker administered (%) |  |
| None | 24 (20.9) |
| Oral | 66 (57.4) |
| Intravenous | 4 (3.5) |
| Oral and intravenous | 21 (18.3) |
| Scan range (mm) |  |
| 120 | 36 cases |
| 128 | 2 cases |
| 130 | 16 cases |
| 140 | 53 cases |
| 160 | 8 cases |
| DLPe (mGy･cm) | 180.9±82.6 |
| Effective dose (mSv) | 2.5±1.2 |
| Tube voltage (%) |  |
| 100kVp | 103 (89.6) |
| 120kVp | 12 (10.4) |
| Tube current (mA) | 573.5±7.9 |
| Heart rate (bpm) | 59.1±0.7 |

DLPe; extended dose length product

**Supplemental Table 3.** Vessel Characteristics

|  | **Overall**  **n = 149** |  |
| --- | --- | --- |
| RCA/LAD/LCX | 33/85/31 |  |
| invasive FFR ≤ 0.80 | 55 (36.9) | 0.83 ± 0.10 |
| CT-FFR_1cm_ | 51 (34.2) | 0.82 ± 0.15 |
| CT-FFR_2cm_ | 63 (42.2) | 0.80 ± 0.17 |
| CT-FFR_lowest_ | 115 (77.8) | 0.63 ± 0.21 |

RCA; right coronary artery LAD; left anterior descending artery LCX; left circumflex branches
